# Vertebral artery terminating in posterior inferior cerebellar artery: A normal variation with clinical significance

**DOI:** 10.1371/journal.pone.0175264

**Published:** 2017-04-10

**Authors:** I-Wen Liu, Bo-Lin Ho, Chien-Fu Chen, Ke Han, Chung-Jung Lin, Wen-Yung Sheng, Han-Hwa Hu, A-Ching Chao

**Affiliations:** 1 Department of Neurology, Taipei Veterans General Hospital, Taipei, Taiwan; 2 Department of Neurology, Kaohsiung Medical University Hospital, Kaohsiung, Taiwan; 3 Department of Neurology, Kaohsiung Municipal Gangshan Hospital, Kaohsiung, Taiwan; 4 Department of Neurology and Neuroscience Center, First Hospital of Jilin University, Changchun, Jilin, China; 5 Department of Radiology, Taipei Veterans General Hospital, School of Medicine, National Yang Ming University, Taipei, Taiwan; 6 Graduate Institute of Clinical Medicine and Department of Neurology, College of Medicine, Taipei Medical University and Hospital, Taipei, Taiwan; 7 Department of Neurology, College of Medicine, Kaohsiung Medical University, Kaohsiung, Taiwan; Universitatsklinikum Freiburg, GERMANY

## Abstract

A vertebral artery (VA) terminating in a posterior inferior cerebellar artery (PICA) is often considered to be a normal variation associated with VA hypoplasia. We aimed to investigate the clinical significance of this cerebrovascular variant. A total of 80 patients with clinically evident cerebrovascular events in posterior circulation were examined by duplex sonography and magnetic resonance angiography (MRA). Eighty healthy subjects who had MRA check-up were recruited as controls. PICA termination of the VA (PICA-VA) was identified as the VA not communicating with the basilar artery (BA) but ending into a PICA. We compared the prevalence of PICA-VA and associated hemodynamic parameters between the patients with and without PICA-VA, and investigated their relationships with VA hypoplasia. The prevalence of PICA-VA was higher in the patient group than in the controls (18.7% vs. 6.3%, p = 0.015). Most measurements (73.3%) of PICA-VA did not fit the criteria of VA hypoplasia. In comparison with the non-PICA-terminating group, the PICA-VA has a smaller diameter (3.7 ± 0.7 mm vs. 3.0 ± 0.5 mm, p < 0.001), lower mean velocity (241 ± 100 mm/sec vs. 164 ± 88 mm/sec, p < 0.01), and higher pulsatility index (1.3 ± 0.5 vs. 1.9 ± 0.6, p < 0.001). Moreover, a smaller diameter of the BA (3.2 ± 0.5 mm vs. 2.5 ± 0.9 mm, p = 0.004) and the posterior cerebral artery (PCA) (2.0 ± 0.1 mm vs. 1.6 ± 0.1 mm, p = 0.006) were also noted in the PICA-VA group. The higher prevalence of PICA-VA in the patient group with smaller diameter of VA, BA and PCA reflected its clinical significance, suggesting that PICA-VA may have a detrimental impact on cerebral hemodynamics. However, the sample is small, and further studies are needed with larger sample size for confirmation.

## Introduction

The vertebral arteries (VAs) are typically arising from the subclavian artery, ascending in the neck and uniting to form the single basilar artery (BA). Anatomical variations of VA may be present as complete or partial duplication, asymmetry due to unilateral hypoplasia, or termination into its principal branch, the posterior inferior cerebellar artery (PICA) [1]. PICA termination of VA (PICA-VA) is occasionally found on routine brain magnetic resonance angiography (MRA); however, only a few studies to date have reported the prevalence of this vascular variant. A review on vertebrobasilar ischemic strokes found that three of 39 patients (7.7%) had PICA-VA by pathological or angiographic examinations [2]. In a normal population, it was estimated that two percent of people had PICA-VA on the right side [3]. Nonetheless, little is known about the clinical relevance of PICA-VA in the high risk group with cerebrovascular diseases.

Anatomical variations of VA could be simultaneously associated with PICA-VA and VA hypoplasia. A hypoplastic VA which terminates into the PICA is susceptible to cervical compression, and may exhibit syndrome of rotational VA occlusion on the same side as the precipitating horizontal head rotation [4]. In addition, VA hypoplasia has been considered to be a possible predisposing factor for posterior circulation stroke [5]. In this study, we assessed hemodynamic parameters of PICA-VA by duplex sonography and MRA. We aimed to evaluate the clinical significance of PICA-VA, and to investigate its association with VA hypoplasia.

## Materials and methods

### Patient enrollment

Patients with previous stroke or transient ischemic attack in posterior circulation territories were recruited from Taipei Veterans General Hospital. Cerebral vasculature was examined in all patients by duplex sonography and cranial magnetic resonance imaging (MRI)/MRA. For comparison of the frequency of PICA-VA, healthy subjects matched for age and sex were recruited from people receiving physical check-ups and who had no history of neurologic signs or symptoms as the control group. They received head and neck MRI/MRA as well for comprehensive survey of cerebral vasculature.

PICA-VA was defined as that the distal VA not communicating with the BA but ending into an ipsilateral PICA, based on non-contrast 3D time-of-flight MRA. The diagnostic criteria of VA hypoplasia, based on contrast-enhanced MRA of the second segment of the extracranial VA was a diameter < 2.5 mm with diameter asymmetry (side to side difference) ≥ 1.2 mm [6].

Fetal type posterior cerebral artery (fetal-PCA) included all of the PCAs which arose from the terminal internal carotid artery without being connected to the BA or having P1 hypoplasia, meaning the diameter of P1 was smaller than that of the posterior communicating artery [7].

Identification of intertransverse segments of VA (V2) hypoplasia was based on contrast-enhanced MRA. The VA diameters of ipsilateral (n = 15), contralateral (n = 15) of patients with PICA-VA, and that of patients without PICA-VA (n = 130) were measured and averaged respectively. Identification of PICA-VA and fetal-PCA were based on head MRI, including T2, FLAIR, DWI, ADC maps, and 3D-TOF. The MRI of extra- and intra-cranial vessels was interpreted blindly by one neuroradiologist and one neurologist independently. We obtained the peak systolic flow velocity (PSV), the end-diastolic flow velocity (EDV) by duplex sonography first. Mean velocity (MV) was calculated as EDV+(PSV-EDV)/3, and the pulsatility index (of Gosling) as (PSV-EDV)/MV. Flow volume was time-averaged velocity × area × 60 seconds. Taipei Veterans General Hospital’s institutional review board approved the study protocol, and a written informed consent was obtained from all participants.

### MRI and MRA study

#### Extracranial vessels

Contrast-enhanced MRA were performed on a 1.5 T scanner (Sigma, CV/i, GE Medical Systems, Milwaukee, Wisconsin) and intravenous gadolinium (0.1 mmol/kg) was administered to all test subjects. The two runs including native and arterial-predominant phase were acquired to obtain the subtraction imaging. The acquisition parameters were: TR 5.71ms, TE 1.2 ms, flip angle 45°, acquired, 34 slices, slice thickness 4 mm with a slice overlap of 3.2 mm, field of view 320 x 320 mm, matrix size 256 x 192).

#### Intracranial cerebral vessels

Time of flight MRA for intracranial arteries were performed with the following parameters: TTR 39 s TE 6.9 s, 224× 224 matrix interpolate to 512 × 512, and 22 × 18 cm2 FOV, 20° FA, 1.8 mm slice thickness interpolate to 0.9 mm slice interval. Spatial saturation pulse was employed at a small distance to the superior end of the 3D slab in order to suppress venous flow. To reduce saturation effects, source images were generated using spoiled gradient recalled acquisition in the steady state. Post processing was performed using a maximum intensity projection with the horizontal and vertical rotation (9.5°–15°) of manual-separated vertebrobasilar system. The diameter of VA was determined by the largest diameter of V2 segment.

### Duplex ultrasound

We used a Philips SD 800 system with a 7.5-MHz linear transducer. The technician was blind to the subjects’ status and responsible for all examinations. The protocol in this study has been established in our previous research [8]. The peak systolic and end-diastolic velocities, and mean velocity were recorded. All measurements were performed twice, and the values averaged.

### Statistical analysis

All parametric results are expressed as mean±SD, median, range (for continuous variables) or percentage (for discrete variables). Chi-square test was used for comparisons of the history of stroke risk factors and frequency of PICA-VA, and all the baseline variables were included in the logistic regression multivariable model for testing if the PICA-VA is still an independent relative risk for stroke. The proportion of PICA-VA or fetal–PCA were tested with Chi-square test. PICA-VA and non-PICA-VA groups were regarded as two independent samples, and the Wilcoxon rank-sum test was used to determine whether there was statistically significant difference in the continuous data of MRI and ultrasound between the two groups. And Wilcoxon signed-rank test were used for within group comparison of bilateral VA in patients of PICA-VA. Two-sided p values of less than 0.05 were considered to indicate statistical significance. All analyses were performed with SAS 9.2.

## Results

One hundred and four consecutive patients, who admitted in the Department of Neurology at Taipei Veterans General Hospital from January 1^st^ 2011 to December 31, inclusive of 91 males and 13 females, participated in the study. Among these patients with clinically evident cerebrovascular events in posterior circulation, we excluded 24 patients with moderate to high-grade VA stenosis (greater than 50%) because the flow velocity and angiographic appearance of downstream vessels would be markedly interfered by the stenotic lesions. Therefore, 80 patients (67 males and 13 females, mean age 71.17 ± 10.2 years, range 28–88 years) were enrolled for subsequent analysis. For comparison of the frequency of PICA-VA, 80 healthy subjects who had MRA check-up were recruited as controls.

The demographic data of patients and age-/gender-matched controls were shown in Table 1. There were significant differences in the vascular risk factors, such as hypertension, diabetes mellitus and hyperlipidemia, between patients and controls. In addition, twenty participants were identified as having PICA-VA, including 15 patients and 5 normal controls. The prevalence of PICA-VA was significantly higher in the patients group than in normal controls (18.8% vs. 6.3%, p = 0.015). The difference in PICA-VA prevalence between groups remained statistical significance (p = 0.0465) after adjusted for history of hyperlipidemia, hypertension and diabetes mellitus. Fourteen (70%) of those with PICA-VA were on the right side.

**Table 1:** Demographic data, prevalence of PICA-VA and the cardiovascular risk factors in patients and controls.

	Patients	Control	P value
	(n = 80)	(n = 80)	
Age	70.7±10.7	70.7±10.7	
	(35–87)	(35–87)	
Gender (M/F)	67/13	67/13	
PICA-VA	15 (19%)	5 (6%)	0.0168
Coronary artery disease	12 (15%)	5 (6%)	0.0725
Hyperlipidemia	30 (38%)	10 (13%)	0.0003
Hypertension	64 (80%)	29 (36%)	<0.0001
Diabetes Mellitus (DM)	39 (49%)	6 (8%)	<0.0001

PICA-VA = posterior inferior cerebellar artery (PICA) termination of vertebral arteryThe difference in PICA-VA between groups remained statistical significance (p = 0.0465) after adjusted for history of hyperlipidemia, hypertension and DM.

Table 2 shows the vessel diameter by MRA and flow profiles by ultrasound. For VAs, the mean diameter of V2 segment in patients with PICA-VA was significantly smaller compared to non-PICA-terminating group (p < 0.0001), but only 4 (26.7%) of 15 patients with PICA-VA fulfilled VA hypoplasia according to the MRA criteria. The end-diastolic velocities (p<0.001) and mean velocity (p < 0.01) of VA was significantly lower, and pulsatility index (p < 0.0001) was significantly higher in the PICA-VA group among patients. For BAs, the patients with PICA-VA had a significantly smaller mean diameter (p = 0.004). Furthermore, the mean diameter of PCA was significantly smaller in the patients with PICA-VA (p = 0.006). The prevalence of fetal-PCA was not different between the patients and normal controls (20% vs. 18.7%, p = 0.272).

**Table 2:** The vascular measurements in patients with ischemic events in posterior circulation.

	Patients with PICA-VA (n = 15)		Patients without PICA-VA (n = 65)	*P* value
	PICA	Contralateral		
**Vertebral artery**	(n = 15)	(n = 15)	(n = 130)	
Diameter of V2 (mm)	3.0 ± 0.5	3.9 ± 0.6 †	3.7 ± 0.7 ‡	
	(2.8,2.3–4.3)	(3.8,2.7–4.9)	(3.7,1.8–6.7)	
Peak systolic of VA(mm/s)	360±179	490±213	440±166	
	(290,150–800)	(460,230–1100)	(420,60–1010)	
End diastolic of VA(mm/s)	67±50	169±97†	142±80†	
	(60,0–160)	(160,80–470)	(135,0–490)	
Mean velocity of VA(mm/s)	164 ± 88	276 ± 129*	241 ± 100*	
	(137,70–373)	(267,132–684)	(243,40–616)	
Pulsatility index of VA	1.9 ± 0.6	1.2 ± 0.4 †	1.3 ± 0.5 ‡	
	(1.7,1.1–3.0)	(1.1,0.7–2.1)	(1.2,0.4–3.0)	
**Basilar artery**				
BA diameter (mm)	2.5 ± 0.9		3.2 ± 0.5	0.004
	(2.8,0.5–3.4)		(3.2,2.2–4.1)	
Peak systolic of BA(mm/s)	832±494		757±419	0.326
	(700,250–2250)		(620,280–2120)	
End diastolic of BA(mm/s)	341±283		315±208	0.773
	(280,90–124)		(250,100–1020)	
Mean velocity of BA(mm/s)	504 ± 350		462 ± 272	0.445
	(402,143–1577)		(360,160–1313)	
Pulsatility index of BA	1.1 ± 0.2		1.0 ± 0.3	0.349
	(1.1,0.6–1.4)		(1.0,0.4–1.7)	
**Posterior cerebral artery**				
PCA diameter (mm)	1.6 ± 0.1		2.0 ± 0.1	0.006
	(1.6,0.9–2.3)		(2.0,0.8–3.1)	

PICA-VA, PICA termination of the VA

Data were expressed as mean±SD; (median, range) for continuous variables or percentage for discrete variables.

*: compare to PICA, p<0.01

†: compare to PICA, p<0.001

‡: compare to PICA, p<0.0001

## Discussion

The main finding of this study is that the prevalence of PICA-VA in patients with clinically evident cerebrovascular events in posterior circulation was significantly higher than that of healthy controls. This suggests that PICA-VA may play an important role in the occurrence of stroke or transient ischemic attack in the patient group. Because of the fact that higher pulsatility index and lower mean flow velocity have been reported in aged people, patients with white matter disease, patients of dementia and patient with traumatic head injury [9–13], thus, we consider the flow profile with higher pulsatility index and lower mean flow velocity as “unfavorable hemodynamics”. Our study revealed PICA-VA was associated with unfavorable hemodynamics, such as significant lower mean flow velocity and higher pulsatility index in the ipsilateral VA, which may explain why PICA-VA can be a risk factor for cerebrovascular disease.

Contrary to the previous belief that PICA-VA has a strong association with VA hypoplasia, we found 73.3% of our patients with PICA-VA did not fit the criteria of VA hypoplasia [14], even though it was highly associated with smaller ipsilateral VA. The mean diameter at V2 segment of PICA-VA was 3.0 ± 0.5 mm, which is higher than the most commonly used definition of VA hypoplasia with VA diameter equal or less than 2.5 mm [14]. In addition, the diameters of BA and PCA were significantly smaller in the PICA-VA group, which may represent hypogenesis of the whole vertebrobasilar system, and further contributed to posterior circulation insufficiency and stroke [7]. However, we found the prevalence of fetal-PCA, another normal cerebrovascular variant, did not increase in patients with PICA-VA.

Our results were compatible with the previous study. Saito et al. examined 128 patients with stroke or atherosclerotic cerebral vessels of which most ischemic events were in the anterior circulation, and they found the VA diameter of the PICA-VA group was significantly smaller compared to the control group (2.6 ± 0.4 mm vs. 3.8 ± 0.7 mm; p < 0.001) [15]. Most of their patients with PICA-VA were not within the range by the definition of VA hypoplasia. Meanwhile, higher asymmetry and resistance index, and lower mean flow and end diastolic velocity were also noted in the PICA-VA group than in the control group. They found the prevalence of PICA-VA was 6% (15 in 240 vessels), which is comparable to the control group but is less than the patients with ischemic events in the posterior circulation in our study.

The VA hypoplasia is thought of as one of the risk factors of ischemic events in the posterior circulation [16]. According to our findings, PICA-VA is also suggested to be a contributor for ischemic events in the vertebrobasilar system, even most of which do not fit with VA hypoplasia. Thus knowing whether or not high-risk patients have PICA-VA simply by non-invasive ultrasound examination has a predictive value for clinical physicians. We propose that PICA-VA should be taken into consideration if a doppler ultrasound reveals a mildly smaller VA with higher pulsatility index. For patients with acute stroke, PICA-VA by ultrasonographic investigation could serve as an accessible evaluation of atherosclerotic or occlusive lesions of the posterior circulation.

Rotational vertebral artery syndrome (RVAS), also known as bow hunter’s syndrome, is rare but clinically important [17]. It is characterized by recurrent paroxysmal vertigo, tinnitus, ataxia, and nystagmus elicited by head rotation. Some researchers presumed that the mechanism of RVAS is transient compression of the dominant VA by head rotation causing ipsilateral labyrinthine excitation in patients with stenosis or hypoplasia of contralateral VA [18]. However, RVAS is not only caused by compression of a dominant VA with stenosis or hypoplasia of a contralateral VA, but also by compression of a non-dominant PICA-VA, which may lead to even more severe sequelae. Noh et al. demonstrated one case with atypical RVAS due to compression of the non-dominant VA terminating in a PICA. Cerebral angiography revealed complete occlusion of the non-dominant VA at the level of the C1–2 junction when the head was rotated to the other side and transient ischemia of the inferior cerebellum or lateral medulla may explain this phenomenon [19]. Yeh et al. also reported a case with persistent vertigo, double vision and unsteady gait caused by occlusion of a non-dominant left VA ending in a PICA. Brain MRI confirmed a left lateral medullary infarction and Wallenberg’s syndrome is caused by compression of a non-dominant left VA ending in a PICA [20]. Mehalic et al. also described a 30-year-old male with acute infarcts of the bilateral brainstem immediately after chiropractic manipulation. The patient was found to have a small left VA terminating in a PICA [21]. Therefore, in addition to RVAS by compression of the dominant VA, small non-dominant PICA-VA is also contributable to vertebrobasilar ischemia during neck motion. Furthermore, PICA-VA was even more vulnerable for vertebrobasilar ischemia if it is along with other risk factors such as atherosclerosis, osteoarthritis, and vertebral ligament laxity [22].

This study had some limitations. First, this study was retrospective with a small number of cases. Though we found a significantly higher frequency of PICA-VA in the patient group than the control, the sample is not large enough which needs to be further studied with larger sample size for confirmation. Second, the PICA-VA group may have included asymptomatic isolated V4 occlusion as well; however, it was difficult to distinguish asymptomatic isolated V4 occlusion from PICA-VA according to the quality of current MRI. Third, we did not have ultrasound data for the control group, and we did not measure the diameter of intracranial cerebral vessels for the control group due to the quality of MRA which was not good enough for us to access with confidence. Fourth, though rotational vertebral artery syndrome is clinically important, and we had the patients with rotational syndrome, but we did not calculate the total number of patients with rotational syndrome in this study. Last, a major limitation is that some important risk factors for stroke such as atrial fibrillation, and in particular atherosclerosis of the vertebrobasilar circulation and intracranial artery stenosis of the BA and VA were not included as risk factors for posterior circulation ischemia in this multivariate analysis between both groups. Further prospective studies with a larger series of patients may be needed to confirm the clinical significance of PICA-VA and its long-term outcomes.

## Conclusions

Although PICA-VA has been considered as a normal variation, our analyses showed its important clinical significance. PICA-VA was associated with a smaller ipsilateral VA, BA and PCA, and it may represent hypogenesis of the whole vertebrobasilar system. Furthermore, we identified the unfavorable hemodynamics and the significantly higher prevalence of PICA-VA in patients with cerebrovascular events in posterior circulation. PICA-VA can be a negative hemodynamic contributor in posterior cerebral circulation, and may be attributable to the occurrence of stroke or transient ischemic attack. However, the sample is small, and further studies are needed with larger sample size for confirmation.
